# Development and evaluation of a portable and soft 3D-printed cast for laparoscopic choledochojejunostomy model in surgical training

**DOI:** 10.1186/s12909-023-04055-0

**Published:** 2023-01-31

**Authors:** Jianfu Xia, Jinlei Mao, Hao Chen, Xiaodong Xu, Jing Zhang, Jin Yang, Zhifei Wang

**Affiliations:** 1 Department of General Surgery, The Second Affiliated Hospital of Shanghai University (Wenzhou Central Hospital), Wenzhou, 325000 China; 2 Soochow University, Suzhou, 215000 China; 3 General Surgery, Cancer Center, Department of Hepatobiliary & Pancreatic Surgery and Minimally Invasive Surgery, Zhejiang Provincial People’s Hospital, Hangzhou, 310000 China; 4 The Second Clinical Medical College, Zhejiang Chinese Medical University, Hangzhou, 310000 China; 5 College of Materials Science and Engineering, Zhejiang University of Technology, Hangzhou, 310014 China; 6 Department of General Surgery, Sir Run Run Shaw Hospital, Zhejiang University School of Medicine, Hangzhou, 310000 China

**Keywords:** 3D printing, Medical education, Training, Choledochojejunostomy, Model

## Abstract

**Background:**

Laparoscopic choledochojejunostomy (LCJ) is an essential basic skill for biliary surgeons. Therefore, we established a convenient and effective LCJ 3D printing model to evaluate whether the model could simulate the actual operation situation and determine its effectiveness and validity in surgical training.

**Methods:**

A 3D printing dry laboratory model was established to simulate LCJ. The face and content validity of the model were evaluated by six experienced biliary surgeons based on 5-point Likert scale questionnaires. A total of 15 surgeons with different levels of experience performed LCJ on the model and evaluated the structural validity of the model using the objective structured assessment of technical skills (OSATS). Simultaneously, the operation time of each surgery was also recorded. A study was also performed to further evaluate the learning curve of residents.

**Results:**

The operating space score of the model was 4.83 ± 0.41 points. The impression score of bile duct and intestinal canal was 4.33 ± 0.52 and 4.17 ± 0.41 points, respectively. The tactile sensation score of bile duct suture and intestinal canal suture was 4.00 ± 0.63 and 3.83 ± 0.41points, respectively. The OSATS score for model operation in the attending group was 29.20 ± 0.45 points, which was significantly higher than that in the fellow group (26.80 ± 1.10, *P* = 0.007) and the resident group (19.80 ± 1.30, *P* < 0.001). In addition, there was a statistical difference in operation time among surgeons of different experience levels (*P* < 0.05). Residents could significantly improve the surgical score and shorten the time of LCJ through repeated training.

**Conclusions:**

The 3D printing LCJ model can simulate the real operation scenes and distinguish surgeons with different levels of experience. The model is expected to be one of the training methods for biliary tract surgery in the future.

**Supplementary Information:**

The online version contains supplementary material available at 10.1186/s12909-023-04055-0.

## Background

Choledochojejunostomy (CJ) is the most commonly used surgical method in treating biliary surgical diseases, as well as the common surgical regimen in treating malignant biliary obstruction. LCJ is an important skill, which is characterized by complexity, difficulty, challenge, and long learning curve. Lack of skilled operation experience may lead to postoperative cholangitis, bile duct stenosis, bile leakage, peritonitis, and even death [1–3]. During the current Covid-19 pandemic, the number of patients and operations has decreased substantially, which is reflected in the training and teaching of surgical residents [4, 5]. To alleviate this problem, surgical societies, including the Society of American Gastrointestinal and Endoscopic Surgeons (SAGES) and the American College of Surgeons (ACS) are all in support of surgical simulation [6–8]. A recent system overview subdivides simulation based training tools into four categories: virtual reality, wet laboratory (animal organs and corpse models from animals or humans), dry laboratory (synthetic models), and E-Learning [9]. It has been proved that clinical training before surgical intervention can provide safe and effective training for surgical residents, so that trainees can rapidly acquire and maintain surgical skills. At the same time, the acquired operative skills can be directly transferred to the actual surgical environment [10–12].

Novice surgeons often face many challenges in their surgical training. The virtual training platforms can provide an immersive experience, which are conducive to the familiarity with surgical procedures. However, these platforms are expensive, lack tactile feedback, and are not widely available [13]. Although the training of animal organs or animals and human cadavers has realistic advantages, and surgeons have widely accepted its education and training value, there are still some limitations, including high cost, availability, non-repeatability, risk of infectious diseases, and potential ethical issues [14, 15]. Many residents support the use of dry laboratory model at home, which is simple, convenient, and inexpensive, and enables trainees to acquire basic laparoscopic skills, including laparoscopic suture, knotting, and coordination training, but does not simulate advanced operation [16].

With the availability of advanced materials and printing techniques, 3D printing technology has been widely used in the medical field, especially in anatomy education and surgical training, among which 3D printing technology has shown promising results and new applications [17]. Several randomized controlled trials in multiple surgical fields have demonstrated that 3D printing model can simulate relevant operations, thus acquiring early skills and carrying out advanced skill training [18–22]. Despite the wide application of 3D printing in various surgical fields, there remain a lack of relevant application in the surgical training of LCJ.

We described portable and soft model created by 3D printing for dry laboratory training in LCJ. Through the establishment of 3D printing, the model can be used in the dry laboratory of LCJ. This model included a liver with an embedded bile duct and a section of intestinal canal, which can be used for visualization, instrumentation, and laparoscopic anastomosis. Experts in the field of biliary surgery can evaluate the face and content validity of the model, and whether the model can simulate the real surgical situations, distinguish different levels of surgeons, share our experience, reduce learning costs and help surgeons improve their surgical skills.

## Methods and materials

### Participants

This study invited six surgical experts from biliary surgery center of Zhejiang Provincial People’s Hospital and Wenzhou Central Hospital to evaluate the face and content validity of the 3D printing model. All six experts performed more than 10 cases of LCJ in the previous year. At the same time, 15 surgeons from the biliary surgery center were also invited to participate in the structural evaluation of the model.

### 3D-printed dry lab LCJ model production

Anonymized Digital Imaging and Communication in Medicine files were obtained using Mimic 23.0 system from 3D computed tomography scans of disease-free human body to extract/reconstruct the anatomical models of liver, bile duct, and small intestine. The extracted STL file was repaired with Magic24 to obtain the sealing structure. Then, OBJ files were exported from Magic 24 and imported to Zbush for further modification. Mold designations were completed by NX 1899 and either positive mold or negative mold was designed depending on the shape of the organs. The STL files of the designed mold were imported to Magic 24 for further designation of support structure and positioning. Next, an FDM 3D printer was used to print the mold based on sliced data, and the surface treatment and support structure removal are carried out after printing. The mold cavity was treated with Vaseline to ensure smooth removal of models from the mold after curing. The model was produced via casting. The silica gel was poured into the mold from the vacuum box and cured at 25 °C for 1 h. Finally, the mold was removed to obtain the model after solidification. That printing files are available upon request.

**Fig. 1:**
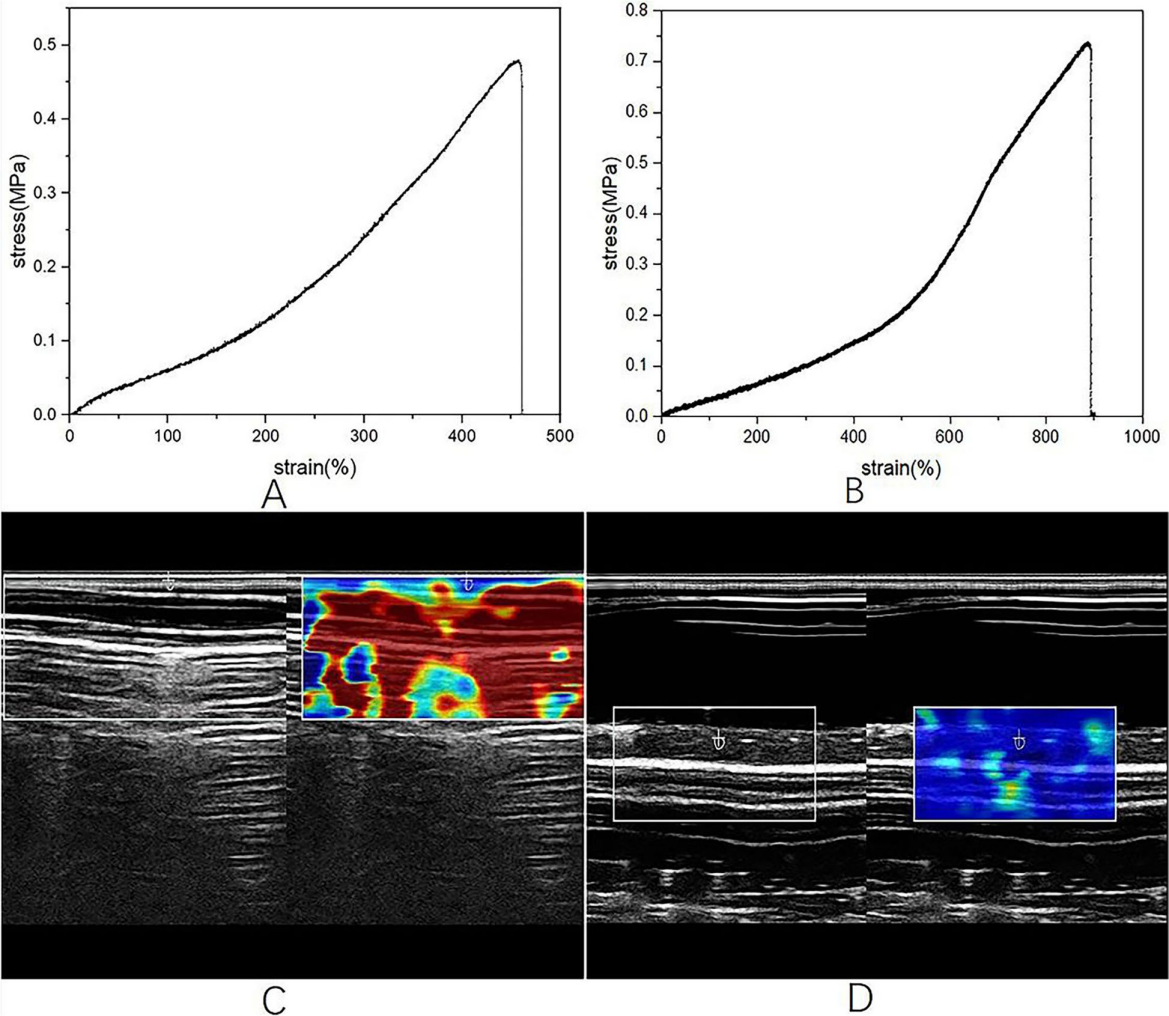
**A** Tensile strength of bile duct (**B**) Tensile strength of small intestine (**C**) Bile duct stiffness (**D**) Small intestine stiffness

The silica gel material used in the bile duct was yellow, with an elastic modulus of 0.16 MPa, and a tensile strength of 0.48 MPa (Fig. 1A). The silica gel material used in the small intestine was red, with an elastic of 0.17 MPa, and a tensile strength of 0.74 MPa (Fig. 1B). The stiffness of model was measured through ultrasound. Ultrasonic Elastic value of bile duct was 1.95 m/s (Fig. 1C), and that of small intestine was 1.47 m/s (Fig. 1D).

**Fig. 2:**
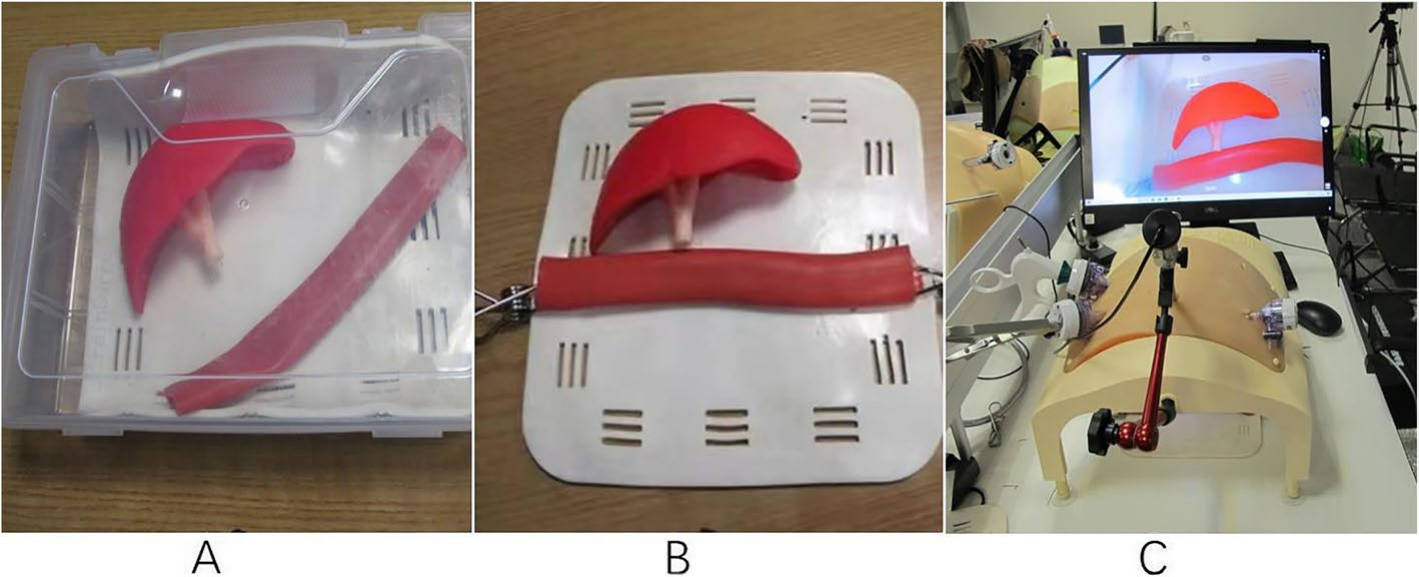
**A** The portable box (**B**) Configuration of 3D-printed models (**C**) The self-designed surgical platform

The box includes (Fig. 2): (1) Liver and its bile duct;(2) Small intestine;(3) Clip for fixing the intestinal canal;(4) The self-printed laparoscopic operation platform that can adjust the height of the platform up and down to simulate pneumoperitoneum, and the silica gel thickness of the abdominal wall skin can be used to place the laparoscopic cannula;(5) Lens fixer, a flexible metal snake bone lens that supports the 30-degree camera, allows surgeons to operate independently. In addition, monitors, sutures, lenses, and laparoscopic instruments were also used in the training.

### Patient and public participation

Neither patients nor the public were directly involved in the design of the current study.

### Model face validity and content validity

Referring to the relevant literature, the face validity and content validity of the model was comprehensively designed [23–25]. The six biliary surgery experts used the 5-point Likert scale (5: Strongly agree; 4: Agree; 3: Neutral; 2: Disagree; 1: Strongly disagree, Additional file 1) to evaluate the model, including the impression, fidelity, texture, appearance, operation space and tactile sensation of the 3D model, as well as its effectiveness for clinical treatment and training.

### Model structure validity

A total of 15 attendings, fellows, and residents were recruited, with five members in each group, and basic information collection tables were issued. All surgeons provided informed written consent. Through pre-recorded video, the explanation and model training operation were performed. The resident group also completed eight-time LCJ training program on the model. The entire operation procedure was captured on video. Our study used a modified edition of OSATS [26, 27] (out of 30 points, Additional file 1). Two experts used OSATS to independently evaluate the recorded video. Respect for tissue, time and motion, instrument handling, flow of operation and forward planning, knowledge of specific procedure and overall performance of attendings, fellows and residents were mainly evaluated. The operation time was recorded at the same time, ignoring the identity of surgeons.

### General information for biliary surgeons

A total of 15 surgeons were invited to participate in the current study. All the surgeons involved in the study were right-handed. There were significant differences in the working year of surgeons in the three groups (14.20 ± 1.64vs 6.40 ± 1.14vs 2.80 ± 0.45, respectively; *p* < 0.001). There were significant differences in the number of cases of LCJ performed by the three groups of surgeons as the chief surgeon (*P* = 0.003) and the number of cases of LCJ performed as the first assistant (*P* = 0.007). There were no significant differences in the use of surgical simulation tools among the three groups (Table 1).

**Table 1:** General information of participants

	Attendings (*n* = 5)	Fellows (*n* = 5)	Residents (*n* = 5)	*P*-value
Age	42.80 ± 0.84	36.40 ± 1.14	28.20 ± 0.84	< 0.001*
Years of working	14.20 ± 1.64	6.40 ± 1.14	2.80 ± 0.45	< 0.001*
Gender (M/F)	5/0	5/0	4/1	1.000
Hand dominance (R/L)	5/0	5/0	5/0	–
Cases of LCJ as lead surgeon				0.003
0	0/5	3/5	5/5	
1–10	1/5	2/5	0/5	
≥ 10	4/5	0/5	0/5	
Cases of LCJ as first assistant				0.007
0	0/5	0/5	3/5	
1–10	0/5	1/5	2/5	
10–30	0/5	1/5	0/5	
≥ 30	5/5	3/5	0/5	
Box training experience	2/5	3/5	5/5	0.253
VR experience	1/5	2/5	1/5	1.000
3D printing model experience	0/5	0/5	0/5	–
Ex vitro animal experience	2/5	2/5	0/5	0.442
Live animal experience	2/5	1/5	0/5	0.712

*P < 0.001

*Note*: *M* male, *F* female, *VR* virtual reality, *LCJ* laparoscopic choledochojejunostomy

### Surgical procedure

**Fig. 3:**
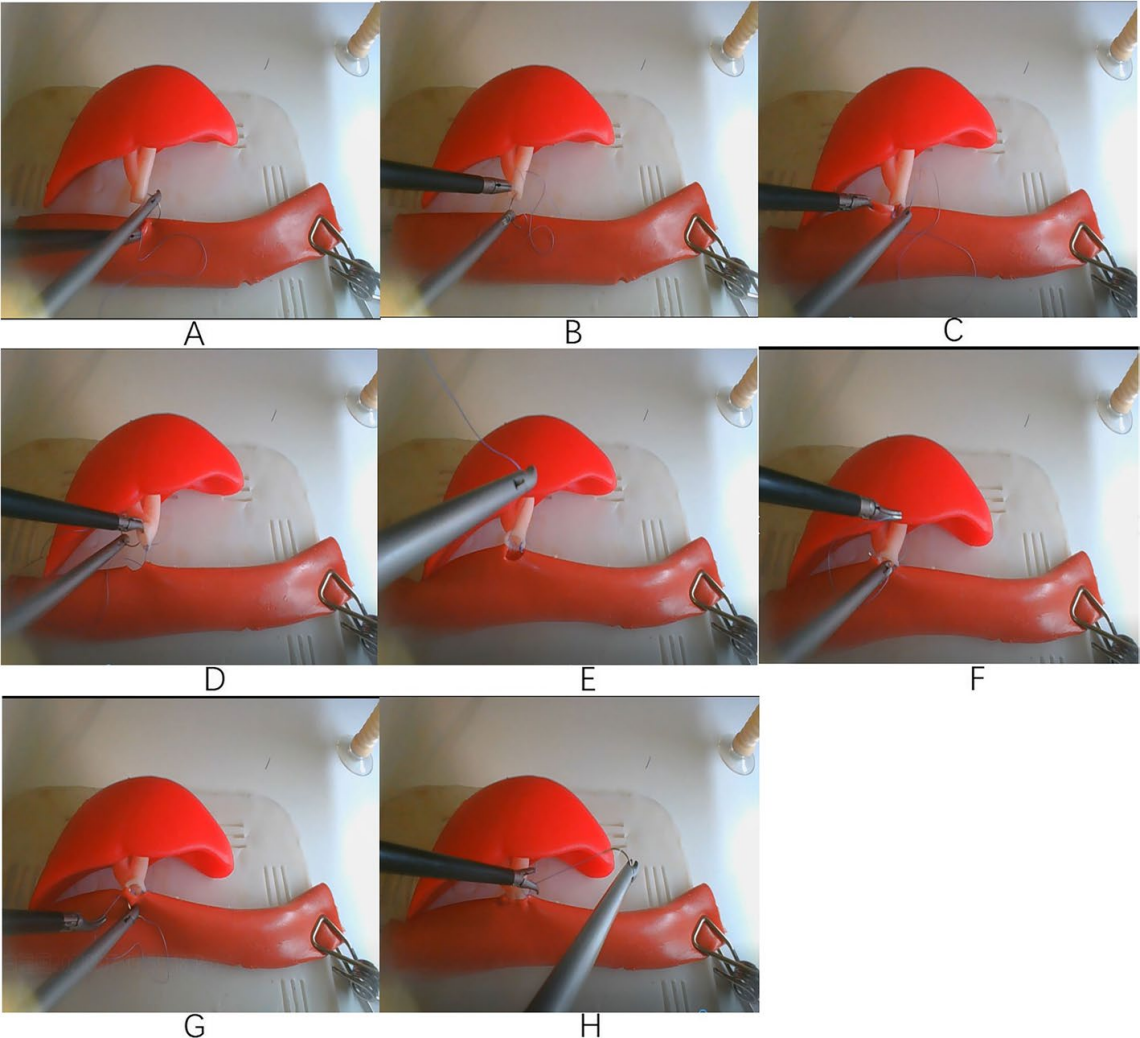
LCJ instructions for 3D printing model **A **The first needle was inserted into the left lateral wall of the intestine. **B** The first needle was inserted into the left lateral wall of the bile duct. **C** The second needle was inserted into the posterior wall of the intestine from inside to outside. **D** The second needle was inserted into the posterior wall of the bile duct from outside to inside. **E** The posterior wall of the intestine had been anastomosed to the posterior biliary ductal wall. **F** The needle was pulled out at the right lateral wall of the intestine. **G** The needle was inserted into the anterior wall of the intestine from outside to inside. **H** Continuously suture the anterior wall of the intestine and the bile duct to complete the anastomosis

Laparoscopic end-to-side anastomosis of bile duct and small intestine with full-thickness continuous valgus suture was used in this study (Fig. 3). 4–0 absorbable suture was used to suture from the left corner of the intestinal opening from outside to inside, and from the left corner of the broken end of the biliary tract from inside to outside. One needle was knotted and the knot was tied outside the anastomosis. The suture needle was sutured into the biliary tract from the left corner of the biliary tract wall, and the second needle was sutured from intestinal tract to the biliary tract. The needle was sutured into the intestinal wall from inside to outside and the needle was sutured into the biliary tract wall from outside to inside. The posterior wall was continuously sutured to the right end. The needle distance and edge distance were maintained at about 2 ~ 3 mm; generally, 5–6 stitches were sutured. When the suture reached the right end of the posterior wall, the suture needle penetrated the suture line of the anterior wall of the anastomosis from the right corner of the intestinal wall. The anterior wall was sutured from right to left, the intestinal wall was sutured from outside to inside and the biliary wall was sutured from inside to outside. Continuous suture was performed until the left end of the anastomosis was knotted with the thread tail to complete the anastomosis.

### Statistics analysis

SPSS version 21.0 (IBM Corp., Armonk, NY, USA) was used for the subsequent data analyses and processing. A comparison of the differences between three groups was performed using the analysis of variance (ANOVA), and multiple comparisons of operative time and operative scores were made using Tukey’s method. Fisher’s exact test was used for intergroup comparisons of count data. The learning curve of the model operation was assessed using the cumulative sum method (CUSUM) and the results were calculated with Excel 2016. Hypothesis tests were all performed using two-tailed probabilities, and the significance level was set at α = 0.05.

## Results

### Face validity score of the model

**Fig. 4:**
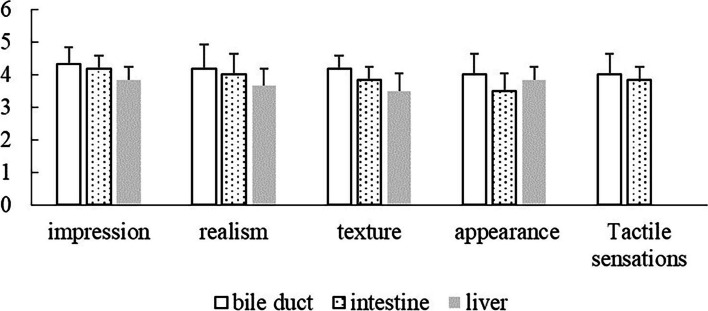
Likert scale results of the face validity evaluation of the various parts of the model

A total of 6 biliary surgery experts were invited to conduct the face validity evaluation for this study (Fig. 4). The operation space score of the model was 4.83 ± 0.41. The impression score of bile duct, intestinal canal and liver was 4.33 ± 0.52, 4.17 ± 0.41 and 3.83 ± 0.41, respectively. The realism score of bile duct was 4.17 ± 0.75, which was higher than the other two parts. The texture score of bile duct was 4.17 ± 0.41, which was higher than the other two parts. The appearance score of bile duct was4.00 ± 0.63, which was higher than the other two parts. The tactile sensations score of bile duct and intestinal canal was 4.00 ± 0.63 and 3.83 ± 0.41, respectively.

### Content validity score of the model

Except that the similarity score between the model and the real operation was less than “agree” (4 points), the evaluation given by all experts was greater than 4 points (Table 2). The rationality of model training and suggestion to use the model in LCJ training have been strongly supported by all experts. The model was considered to be easy to operate, which can reduce the risk of patients, improve the skills of participants, shorten the learning curve, and enhance the operative confidence and training interest.

**Table 2:** Likert scale results of the model content validity

	Mean ± SD
The operation of the model was similarity with actual case.	3.83 ± 0.41
The model was easy to handle.	4.50 ± 0.55
The model was reasonable for LCJ training.	4.67 ± 0.52
The model could shorten learning curve and improve trainee’s skills.	4.83 ± 0.41
The model training reduced the risk for patients.	4.50 ± 0.55
The model could improve interest during training.	4.33 ± 0.52
The model could increase operative confidence.	4.33 ± 0.52
I recommend that the model be used in LCJ training.	4.83 ± 0.41

Data are expressed as mean ± standard variation

### Construct validity of the model

**Fig. 5:**
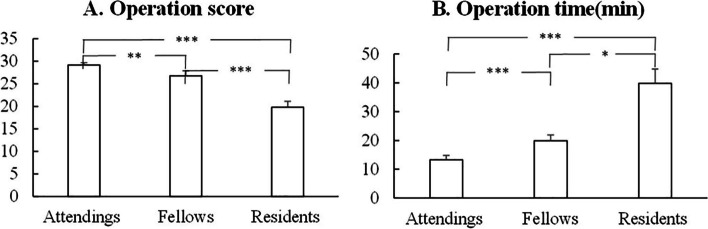
**A** The operation score of the attending group was significantly higher than either that of the fellow group or the resident group; **B** The operation time of the attending group was significantly shorter than either that of the fellow group or the resident group. *P < 0.05, **P < 0.01, ***P < 0.001

There were significant differences in OSATS scores among researchers in the three groups (*P* < 0.01). The score of the attending group was significantly higher than those of fellow group (29.20 ± 0.45vs26.80 ± 1.10, *P* = 0.007) and resident group (29.20 ± 0.45vs19.80 ± 1.30, *P* < 0.001), as shown in Table 3 and Fig. 5A. There were significant differences in the operation time among researchers in the three groups (*P* < 0.05). The operation time of the attending group was significantly shorter than those of fellow group (13.32 ± 1.49 vs19.92 ± 2.02, *P* = 0.016) and resident group (13.32 ± 1.49 vs 39.84 ± 4.88, *P* < 0.001), as shown in Table 3 and Fig. 5B.

**Table 3:** The OSATS score and operation time of the three groups

	Attendings (*n* = 5)	Fellows (*n* = 5)	Residents (*n* = 5)	*P*- value
OSATS score	29.20 ± 0.45	26.80 ± 1.10	19.80 ± 1.30	*<0.001*
Operation time (min)	13.32 ± 1.49	19.92 ± 2.02	39.84 ± 4.88	*<0.001*

* *P*<0.001

### Learning curve assessment

**Fig. 6:**
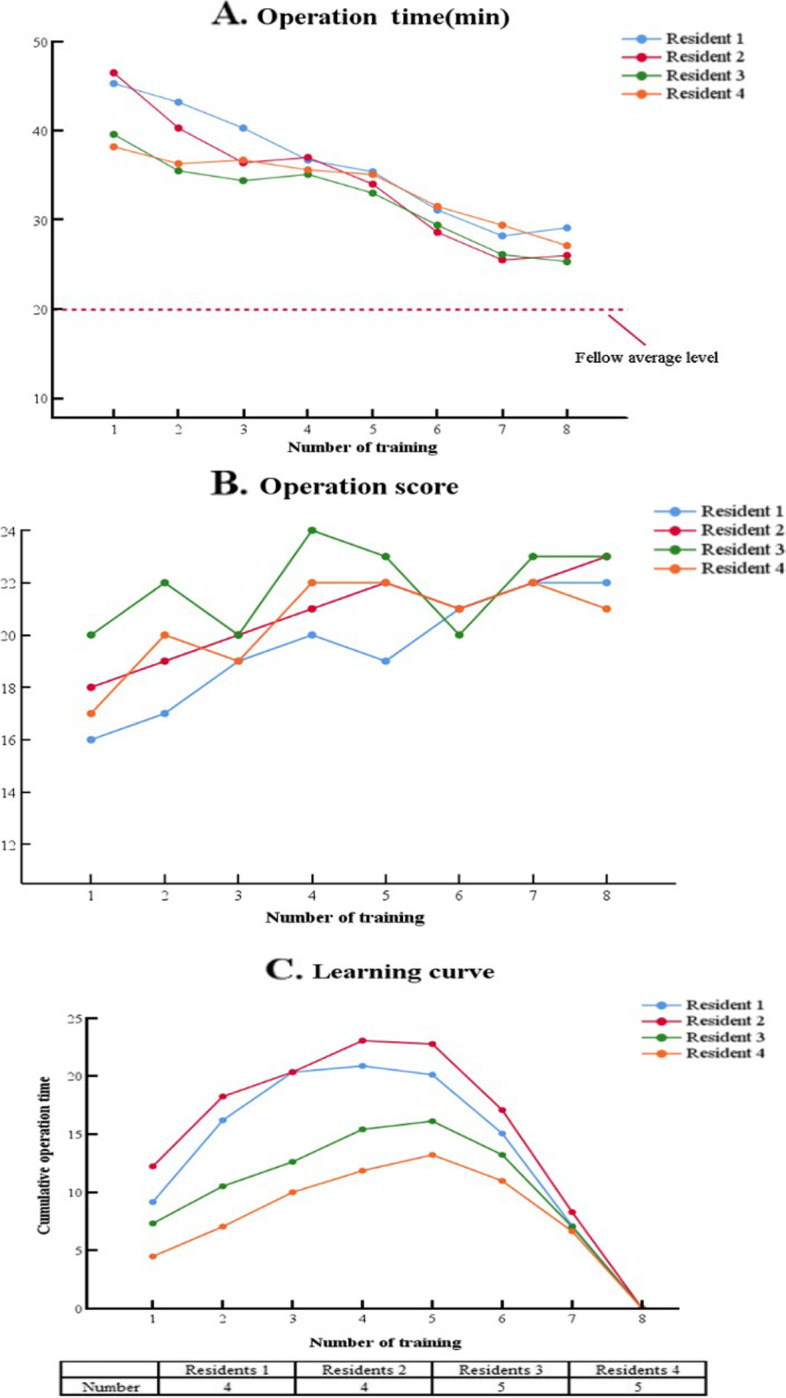
**A** The operation time curve of LCJ **B **The operation score curve of LCJ **C **Learning curve of 4 residents

Four residents were willing were receive a total of eight-time LCJ trainings. The other one resident was unable to participate due to personal reason. The operation times of the four residents were showed in Fig. 6A. The operation scores were shown in Fig. 6B. The average operation scores and operation times of the five fellows were marked with a line. As the number of training increases, the operation times and scores presented gradual progress. It was found that the number of turning points in the learning curve of the residents were 4th case, 4th case, 5th case, and 5th case in the training (Fig. 6C).

## Discussion

Traditional surgical teaching and training methods are facing various pressures. As an alternative educational tool, surgical simulation training figures prominently in the training curriculum [26]. A series of simulation training tools have been developed and verified as effective ones in the medical field, including laparoscopic box trainer and virtual reality simulator [28]. Several randomized controlled trials and systematic evaluations have shown that the technical skills acquired in these simulators can indeed be transformed into skills in the real environment of the operating room [29–33]. Unfortunately, there is still a lack of sufficiently realistic models to simulate the real anatomical structures. The dry laboratory model is made of hard material, which is not conducive to suture. The mechanical simulation of soft tissue has not been optimized, which significantly hinders its application in surgical training (such as suture and cutting). Virtual reality is expensive. It cannot completely simulate the same surgical environment, nor can it include real anatomy and tactile feedback.

3D printing technology has developed rapidly, and become one of the application fields of medicine, biomaterials, tissue engineering and surgery [34, 35].3D printing surgical training model can not only be conducive to preoperative anatomical details of organs, so as to enhance the familiarity with the surgical procedure, improve surgical skills and shorten the learning curve, but also be affordable and have low surgical cost [36, 37].

A recent systemic evaluation showed that 3D models could provide surgeons with equal teaching and training effects as cadaver simulation. With the development of 3D printing and biomaterial technology, they may replace the role of cadaver simulation tools [38]. The six experts in the study believed that the established model can achieve favorable surface validity. They all agreed that the model reconstructed the realistic LCJ and supported its application in LCJ training. In addition, the 3D model can effectively improve surgical skills, self-confidence, learning interest, shorten learning curve and reduce the risks of patients. This indicates that the model can simulate real surgical scenes and play a potential role in surgical training.

According to our previous experience in using 3D printing models for surgical training, we chose LCJ in this study, because CJ is a commonly used choledochojejunostomy in the field of hepatobiliary and pancreatic surgery. Due to the technical difficulty of LCJ, few studies have been conducted to evaluate LCJ. Due to the high error cost, LCJ is not often exposed to surgical resident or fellow. Additionally, variations of the bile ducts pose difficulties that are uneasy to encounter. In this model, we simulated the narrow space by providing the helium part of the liver, as well as typical LCJ situations, such as the anastomosis of left and right hepatic ducts, or the anastomosis to the common hepatic duct. Moreover, the diameter of the bile duct can range from 2 mm to 2 cm. Therefore, the training can be stepwised from easy to difficult. Trainees only needs to take out the model to check the anastomotic stoma. The tube simulating the bile duct is connected to the water pump, which can objectively evaluate and compare the leakage or stenosis of the anastomotic stoma.

Soft silica gel material was used in this study to print the parenchyma of bile duct and small intestine. The materials were continuously modified and adjusted during this period to meet the criteria of surgical training. The model not only simulated the anatomical structure, texture, appearance and tactile sensation of real organs, but also performed the mechanical test. According to literature, the maximum stress of small intestine in human body is 0.9 MPa [39]. The maximum stress of bile duct and small intestine under uniaxial tension is 0.48 MPa and 0.74 MPa respectively, which is close to the maximum stress of human small intestine. All the participants thought bile duct and small intestinal are easy to suture because they are easy to be stretched.

Ultrasonic elastography is a kind of ultrasonic imaging method used to evaluate the hardness of tissue. Through the measurement of strain generated in tissue, the mechanical properties of tissue can be non-invasively evaluated. The detection of 3D printing components through ultrasonic elastography, the mechanical properties of tissues can be evaluated to promote the improvement of materials, so as to make the texture of surgical training materials much closer to the normal human body and improve the training experience. In this study, soft silica gel was used to simulate CJ model and its hardness. The hardness of small intestine was slightly higher than that of normal small intestine. Literature reported that the hardness of normal small intestine was 1.42 ± 0.6 m/s [40]. Currently, there is no relevant literature report on the ultrasonic elasticity of human bile duct. Ultrasonic elasticity of our bile duct model showed its hardness of 1.95 m/s, which was higher than that of small intestine. It was consistent with the fact that the hardness of bile duct is higher than that of small intestine in actual operation. Currently, our team is studying to use hydrogel as a 3D printing material to print CJ model. Its hardness is similar to that of normal CJ. However, the hydrogel is not easy to preserve and is expensive. We believe that with the improvement of 3D printing technology and materials, the issue of tissue preservation and price will be solved. A method of FDM was selected for the model used in this study. This method can quickly and massively manufacture the model by melting the printing mold, and the printing cost of FDM is relatively low.

Evaluation of surgical skills and abilities is an essential aspect of medical education. Through the model operation, the study of operation score and time can be carried out to effectively reflect the operation level of surgeons [41, 42]. A recent systemic review of 24 multidisciplinary studies showed that, through effective evaluation tools, the technical skills of surgeons could be evaluated, which can positively influence the prognosis and outcome of patients [43]. The reliability and effectiveness of OSATS evaluation tool are similar to objective structured clinical examination, which can be widely used in various medical specialties and has been proved to be an effective tool for evaluating the technical ability of surgeons [44, 45]. The surgical score criteria of this study are based on this kind of improved scoring design. Three groups of surgeons were selected in our study to score the construct validity of this model. This model can effectively distinguish the differences in surgical scores and operation time among three groups of surgeons, which further illustrates the training effect of this model, and can distinguish different levels of surgeons, and roughly assess whether biliary surgeons are ready for LCJ surgery.

In the repeated training part, four residents presented gradual progress through repeated. After 8 training sessions, all residents gradually shorten the operation time and improve score, there was still a gap between their levels and the average fellow surgeon level. The turning point in the CUSUM curve indicates a point of trend transition. It was found that the operation time was relatively stable after training for 4–5 cases, and entered a plateau. After more training, we believe that the learning curve of residents should shorten, and their operation time will reach the fellow surgeon levels, even the levels of attending surgeon.

Our study still has several limitations. Firstly, we selected 15 surgeons to perform LCJ on the 3D model, and more experienced surgeons are needed to evaluate the effectiveness of the model. Secondly, it is uncertain whether the skills acquired by the current LCJ model will be directly transferred to the surgical environment, and future validation studies are needed. Thirdly, the current model does not contain blood vessels and simulation of intraoperative bleeding. Our group is currently working on this model for tissue bleeding, which will more perfectly mimic the real situation. Fourthly, soft silica gel material was used to simulate intestinal canal and bile ducts in this study, but its hardness was slightly higher than that of normal tissue. In addition, the intestinal tract and bile duct used in this study were bilayer structures, lacking the multilayered structure of normal tissue. In the future we need to try different materials to achieve better material simulation, such as hydrogel organs and tissues, and compare the effectiveness of their expert evaluation and training. Although the model was anatomically realistic, the training task was limited to laparoscopic sutures. We are working on hydrogels and believe that these technical organizations will be solved in the future.

In conclusion, through this study, we established a new 3D printing model, which can simulate the real LCJ surgical situations, and has similar mechanical properties and hardness to distinguish surgeons with different experience levels. In addition, LCJ model can also help trainees acquire complex surgical skills, shorten the learning curve, reduce risks of patients. Moreover, the training is not limited by time and environment.

## Conclusions

The results of this study on 3D printing LCJ model, the evaluation of face, content and construct validity, have an important influence on surgical training. Our goal is to extend this pattern to biliary surgery and other surgical specialties. Although the model is anatomically real, it is limited to laparoscopic suture, and does not involve cutting and anastomosis parts, such as ultrasonic scalpels and staplers. In future hydrogel studies, we believe that these manipulations will be addressed. In addition, whether the skills acquired from the models will be directly transferred to the surgical setting still needs to be verified in the future.

## Supplementary Information

**Additional file 1: **Pre-LCJ questionnaire.

**Additional file 2.**

## Data Availability

Additional data and materials can be made available upon request to the corresponding author.
