# Supplementary material for: Development and evaluation of a portable and soft 3D-printed cast for laparoscopic choledochojejunostomy model in surgical training
Source: BMC Med Educ. 2023 Jan 31;23:77. doi: 10.1186/s12909-023-04055-0 (PMC9889129; doi:10.1186/s12909-023-04055-0)
Supplement: Supplementary file 1 — Additional file 1: Pre-LCJ questionnaire. [file 12909_2023_4055_MOESM1_ESM.docx]

**APPENDIX 1**

**Pre-LCJ questionnaire**

Name：________________

Demographics

Age：________________ Sex: □Female □Male

Position □ Surgeon

□Attendings

□Fellows

□Residents

Years of surgery experience: ________________

Hand dominance

□Right hand

□Left hand

Model experience

Have you ever learned a laparoscopic choledochojejunostomy using a model? □ Yes

□ No

If yes, what was the procedure or technique you learned?

What type of model had been used？ □ Live animal

□ Ex vitro animal

□The box trainer model

□Computer model

□3D Printing model

□No, have not used a laparoscopic choledochojejunostomy model

Did you think the model is an effective learning tool?

□Yes

□No

□No, have not used a laparoscopic choledochojejunostomy model

**Background and experience**

| Do you had performed LCJ as lead surgeon? | □Yes  □No |
| --- | --- |
| Total number performed (lifetime personal experience) | □0  □ <10  □ >10 |
| Do you had performed LCJ as first assistant? | □Yes  □No |
| Total number performed (lifetime personal experience) | □0  □<10  □10-30  □ >30 |

**Post-LCJ questionnaire**

**Following your hands-on session with this LCJ model**

**Qu.1 Please rate the face validity of the model**

**On a scale of 1 to 5: 1=Strongly disagree, 2 =Disagree, 3 = Neutral, 4 = Agree, 5 = Strongly agree**

| Impression | Overall perception and impression of the model. | 1 2 3 4 5 |
| --- | --- | --- |
| Realism | The fidelity of the model compared to the normal tissue. | 1 2 3 4 5 |
| Texture | The texture of the model compared to the normal tissue | 1 2 3 4 5 |
| Appearance | The similarity score of the appearance of the model and normal tissue. | 1 2 3 4 5 |
| Operative space | The operation space of the training box compared to the real space | 1 2 3 4 5 |
| Tactile sensations | Tactile response during suturing | 1 2 3 4 5 |

**Qu.2 Please rate the content validity of the model**

**On a scale of 1 to 5: 1=Strongly disagree, 2 =Disagree, 3 = Neutral, 4 = Agree, 5 = Strongly agree**

| The operation of the model was similarity with actual case. | 1 2 3 4 5 |
| --- | --- |
| The model was easy to handle. | 1 2 3 4 5 |
| The model was reasonable for LCJ training. | 1 2 3 4 5 |
| The model could shorten learning curve and improve trainee’s skills. | 1 2 3 4 5 |
| The model training reduced the risk for patients. | 1 2 3 4 5 |
| The model could improve interest during training. | 1 2 3 4 5 |
| The model could increase operative confidence. | 1 2 3 4 5 |
| I recommend that the model be used in LCJ training. | 1 2 3 4 5 |

**Qu.3 Please rate Modified Objective Structured Assessment of Technical Skills (OSATS) of model training**

| **Respect for Tissue** | | | | |
| --- | --- | --- | --- | --- |
| 1 | 2 | 3 | 4 | 5 |
| Frequently used unnecessary force on tissues or caused damage by inappropriate use of instruments |  | Careful handling of tissues but occasionally caused inadvertent damage |  | Consistently handled tissues appropriately with minimal damage |
| **Time and Motion** | | | | |
| 1 | 2 | 3 | 4 | 5 |
| Many unnecessary moves |  | Efficient time/motion but some unnecessary moves |  | Clear economy of movement and maximum efficiency |
| **Instrument Handling** | | | | |
| 1 | 2 | 3 | 4 | 5 |
| Repeatedly makes tentative or awkward moves with instruments by inappropriate use of instruments |  | Competent use of instruments but occasionally appeared stiff or awkward |  | Fluid moves with instruments and no awkwardness |
| **Flow of Operation and Forward Planning** | | | | |
| 1 | 2 | 3 | 4 | 5 |
| Frequently stopped operating and seemed unsure of next move |  | Demonstrated some forward planning with reasonable progression of procedure |  | Obviously planned course of operation with effortless flow from one move to the next |
| **Knowledge of Specific Procedure** | | | | |
| 1 | 2 | 3 | 4 | 5 |
| Inefficient knowledge of procedure； looked unsure and hesitant |  | Knew important steps of procedure |  | Demonstrated familiarity of all steps of procedure |
| **Overall Performance** | | | | |
| 1 | 2 | 3 | 4 | 5 |
| Very poor |  | Competent |  | Very good |

1. Martin JA, Regehr G, Reznick R, et al. Objective structured assessment of technical skill (OSATS) for surgical residents. Br J Surg. 1997;84(2):273-278.
2. Birkmeyer JD, Finks JF, O'Reilly A, et al. Surgical skill and complication rates after bariatric surgery. N Engl J Med. 2013;369(15):1434-42.
3. Malas, T., et al., Impact of visualization on simulation training for vascular anastomosis. J Thorac Cardiovasc Surg, 2018. 155(4): p. 1686-1693.e5.

**Qu.4 What was the most challenging part of the procedure?**

**Qu.5 How could the training experience with this model be improved?**
